# Assembly of Microtubules and Actomyosin Rings in the Absence of Nuclei and Spindle Pole Bodies Revealed by a Novel Genetic Method

**DOI:** 10.1371/journal.pone.0000618

**Published:** 2007-07-18

**Authors:** Yinyi Huang, P.T. Tran, Snezhana Oliferenko, Mohan K. Balasubramanian

**Affiliations:** 1 Temasek Life Sciences Laboratory, National University of Singapore, Singapore; 2 Department of Biological Sciences, National University of Singapore, Singapore; 3 Department of Cell and Developmental Biology, University of Pennsylvania, Philadelphia, Pennsylvania, United States of America; Duke University Medical Centre, United States of America

## Abstract

**Background:**

The nucleus and the centrosomes (spindle pole bodies; SPBs in yeast) are believed to play key roles in the organization of various cellular structures, such as the actomyosin ring and microtubules. The ability to generate cells lacking nuclei and centrosomes (SPBs) is key to the elucidation of the role of these structures in various cellular processes.

**Methodology/Principal Findings:**

Here we describe a genetic method, using the *Schizosaccharomyces pombe cdc16*-116 mutant, to reliably and efficiently generate fission yeast cells lacking nuclei and SPBs. We use this approach to show that the assembly of microtubules does not require nuclear associated microtubule organizing centers and SPBs. We also show that actomyosin rings can assemble albeit inefficiently in the absence of nuclei and SPBs.

**Conclusion:**

We conclude that key cytoskeletal elements can be assembled in the absence of nuclei and SPBs. In addition, the approach we describe, taken together with physical approaches such as centrifugation, should facilitate the investigation of the role of the nucleus and SPBs in the assembly and inheritance of various cellular structures and organelles.

## Introduction

The nucleus and the centrosomes (or their functional analog in yeast, the SPBs) are believed to play key roles in induction of the cleavage furrow and organization of microtubules. Whether nuclei, chromosomes, and centrosomes are essential for cleavage furrow and microtubule assembly is an actively debated topic [1]–[9]. Central to ascertaining the role of nuclei and centrosomes (or spindle pole bodies) is the ability to reliably generate cell fragments lacking these structures. In animal cells, microsurgery and laser ablation have been widely used to generate cells lacking nuclei and centrosomes [2], [3], [7]. However, these methods have some limitations. For example, microsurgery might physically damage the cells and fragments of chromosomes and kinetochores might not be inactivated during the procedure.

Cells of the fission yeast *Schizosaccharomyces pombe* have many fundamental properties in common with cells of higher organisms, and therefore have been used to study many cellular processes. In fission yeast, a centrifugation method has been developed to misplace the nucleus away from the cell center. Such cells with a misplaced nucleus, divide to produce a daughter cell with two nuclei and another with none [8]–[10]. The centrifugation method applied in fission yeast, though powerful, is not very efficient. It has been reported that only 4% of cells in a population subjected to centrifugation lacked nuclei [8].

In this study, we have used the *cdc16*-116 mutant, in which cytokinesis remains constitutively active, to generate anucleate cells. The *cdc16*-116 mutant was chosen for the current study for reasons described herein. In *S. pombe*, an actomyosin ring that divides the cell during cytokinesis is assembled upon entry into mitosis and its maintenance and constriction depend on the septation initiation network (SIN), a signaling protein network whose components localize to the SPBs and the cell division site [11]. A key component of the SIN is the GTPase Spg1p, which promotes actomyosin ring assembly and maintenance as well as septation when bound to GTP [12]. Spg1p is regulated by Cdc16p and Byr4p, a two-component GTPase activating protein (GAP) complex [13]. Loss of function of the *cdc16* gene leads to constitutive SIN signaling and multiple waves of actomyosin ring assembly and septation [14] even in the absence of mitosis [15]. As a result, *cdc16-*116 mutant cells shifted to the restrictive temperature during G_1_, S, or G_2_ phases assemble actomyosin rings and un-cleaved septa to produce two connected daughter cells, one of which lacks a nucleus and SPBs [15].

In this study, through the use of anucleate cells lacking SPBs generated in the *cdc16*-116 mutant background, we show that microtubules and actomyosin rings can assemble in the absence of nuclei and SPBs.

## Materials and Methods

### Yeast strains and growth conditions

Yeast strains used in this study are listed in table I. Cells were cultivated and maintained as described previously [16]. To arrest cells in S phase, cells were first treated with 12 mM hydroxyurea (HU; Sigma) for four hours, then treated with the same amount of HU for an additional two hours.

**Table 1:** *Schizosaccharomyces pombe* strains used in this study

Strain	Relevant genotype	Source or reference
MBY286	*cdc16*-116 , *leu1*-32, *ura4*-D18, *ade6*-M210, h+	[14]; Paul Nurse
MBY3007	*cdc16*-116 Pcp1GFP::ura4	This study
MBY2949	*cdc16*-116 Rad24GFP::ura4	This study
MBY4330	*cdc16-*116 Pcp1GFP::ura4 with pPDQ105 (GFP-Atb2p)	This study
MBY2603	*cdc16*-116 Uch2GFP::ura4 Rlc1GFP::leu1	This study

### Fluorescence recovery after photobleaching (FRAP) assay

Photobleaching was performed on a Zeiss LSM 510 laser scanning confocal microscope, equipped with a 63X/1.4NA PlanApo objective lens. An Argon/Krypton laser with ∼10 mW at 488 nm was used for imaging (0.05% power) and photobleaching (100% power). A long pass 505 nm filter was used for visualizing cells.

### Fluorescence and time-lapse microscopy

Staining with DAPI, aniline blue and Alexa 488-conjugated phalloidin was carried out as described previously [17]. Images were captured using an Olympus IX71 microscope. Live cell imaging methods were performed as previously described [18]. To image Rlc1p-GFP, cells were observed on a Zeiss LSM 510 confocal microscope equipped with a 63X/1.4NA PlanApo objective lens. Images were collected in 3D time-lapse mode (0.6 μm step size, 2.5 min intervals). To image GFP-Atb2p, cells were observed on an Olympus 1×71 microscope. Images were collected in 3D time-lapse mode (0.5 μm step size, 15 seconds intervals).

## Results and Discussion

### An efficient genetic method to generate anucleate cells

Recently, a method has been described to produce anucleate fission yeast cells [8]–[10]. This method employs centrifugation to misplace the nucleus in asynchronously growing cells. The ensuing cytokinesis divides the cell into one binucleate and one anucleate daughter cell. However, formation of anucleate cells occurred at low frequencies when the centrifugation method was used. As an alternative, we made use of the temperature sensitive *cdc16*-116 mutant to generate a high number of cells lacking nuclei and SPBs.

**Figure 1:**
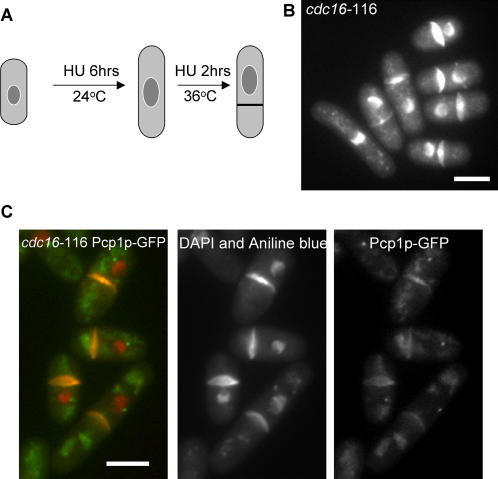
A genetic method to generate anucleate cells. (a) Outline of the strategy to generate anucleate cells. *cdc16*-116 mutant cells were treated with HU for 6 hours at 24°C to arrest cells in interphase. In the presence of HU, cells were shifted to 36°C to inactive Cdc16p, leading to the formation of septa even when the cells were in interphase. The formation of septa divides the cells into two compartments, one of which lacks a nucleus. (b) *cdc16*-116 mutant cells, treated with HU as described above, were fixed and stained with DAPI and aniline blue to visualize nuclei and septa, respectively. Shown are the un-cleaved septa formed in interphase cells dividing the cell into two compartments, one of which lacks a nucleus. (c) Anucleate cells do not contain SPBs. *cdc16*-116 cells expressing Pcp1p-GFP were shifted to the restrictive temperature in the presence of HU, fixed and stained with DAPI and aniline blue to visualize nuclei and septa, respectively. Pcp1p-GFP (green) is associated with nuclei (red) and is not observed in anucleate compartments. Scale, 5 µm.

The general strategy of this method is outlined in Figure 1A. It utilizes the ability of *cdc16* mutant cells to form division septa in interphase, when the cell contains only one nucleus. To enrich the population of cells in interphase, we treated cells with 12 mM hydroxyurea (a drug that prevents DNA synthesis) at the permissive temperature of 24°C. After six hours of incubation with HU, the majority of cells were blocked in interphase due to activation of the S-phase checkpoint. Cells were shifted to the restrictive temperature 36°C in the presence of HU to inactivate the function of *cdc16*, leading to formation of septa. As a result, such cells were divided into two compartments, one with a single nucleus, and the other without a nucleus (Figure 1B). The compartment with a nucleus was usually larger than the one lacking a nucleus. By this method, we increased the percentage of anucleate cells in the population to 56.2%. Among septated cells, 94.6% of cells contained an anucleate compartment.

In fission yeast vegetative cells, the SPB is tightly associated with the nucleus. To examine whether anucleate compartments in *cdc16*-116 mutant cells contain SPBs, we visualized SPBs using the SPB marker Pcp1p-GFP, a calmodulin-binding protein, which is an essential component of SPB [19], [20]. In wild type cells, Pcp1p-GFP associates with SPBs throughout the cell cycle [19]. *cdc16*-116 cells expressing Pcp1p-GFP were treated with HU and then shifted up as described. We found that Pcp1p-GFP associated with nuclei, and Pcp1p-GFP signal was not detected in the anucleate compartment (Figure 1c). This indicates that SPBs are absent in anucleate compartments in *cdc16*-116 mutant cells.

### FRAP revealed distinct nucleate and anucleate compartments in *cdc16*-116 cells upon cytokinesis induced in interphase

**Figure 2:**
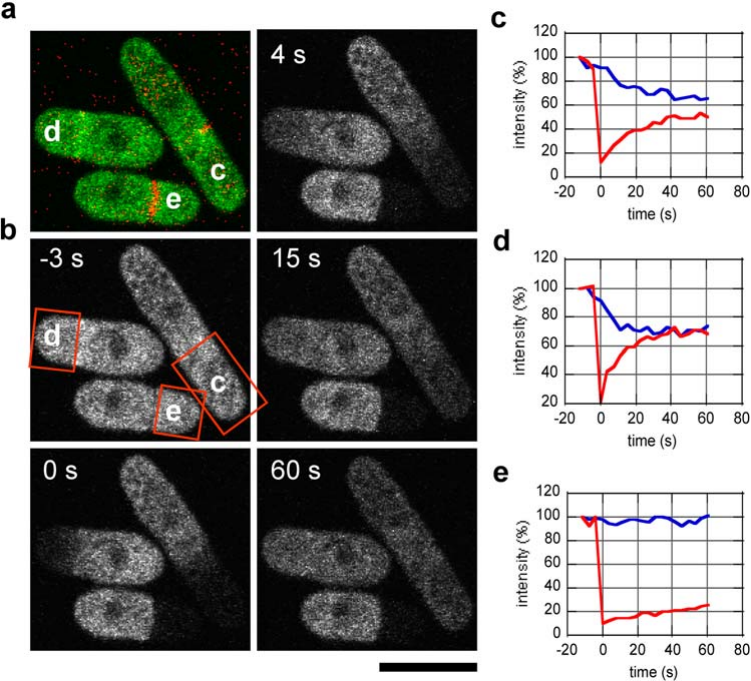
FRAP reveals distinct anucleate cells in the *cdc16*-116 mutant. (a) Merged pseudo-colors image of *cdc16*-116 cells expressing Rad24p-GFP (green) stained with the cell wall tracer aniline blue (red). Temperature sensitive *cdc16*-116 cells were treated with hydroxyurea (HU) for 6 h to arrest cells at S phase, and shifted to the non-permissive temperature of 36°C for 2 h. The three cells shown are in different stages of septation, ranging from early septation (cell c) and mid-septation (cell d) with shared cytoplasm between the two daughter cell halves, to completed septation with a distinct cell wall separating the two daughter cell halves (cell e). (b) Fluorescence recovery after photobleaching (FRAP) was performed on cells at various stages of septation. Cell regions c, d, and e are regions that were photobleached (red dotted boxes). Shown is a time-lapse montage of the behavior of Rad24p-GFP within 60 s after photobleaching. (c, d, e) Normalized fluorescence recovery curves of cell c, d, and e, respectively. Cell c and d showed complete recovery within ∼60 s after photobleaching (□_1/2_ ∼8 s). The unbleached cell halves (blue lines) showed fluorescence decay as a mirror image to the fluorescent recovery of the bleached cell halves (red lines). Cell e showed no significant recovery in the bleached half, and no decay in the unbleached half. Scale, 5 µm.

Although our method generates high numbers of anucleate cells devoid of SPBs, we noted that during repeated rounds of cytokinesis, the nucleate and anucleate compartments remained attached after septation. To determine if the cytoplasm of the anucleate compartment is discontinuous from that of the attached nucleate compartment we performed fluorescence recovery after photobleaching (FRAP) studies. *cdc16*-116 cells expressing Rad24p-GFP, a 14-3-3 protein which resides largely in the cytoplasm [21], were used in these studies. Cells at different stages of septation are clearly distinguished by the cell wall staining with aniline blue (Figure 2a). We performed FRAP on cells early in septation (Figure 2b, cell d), halfway through septation (Figure 2b, cell c), and cells that had completed septation (Figure 2b, cell e). In cells which were in the process of septation, fluorescence recovery (recovery half-time τ_1/2_: ∼8 s) was observed in the bleached half, and the corresponding fluorescent decay was observed in the unbleached half (Figure 2b, c&d). In contrast, in attached cells that had completed septation, no evidence of fluorescence recovery was observed (Figure 2b&e). To establish the limit of our error in scoring cells with completed septa, we performed FRAP on 408 cells which showed completed septa as judged by aniline blue staining as well as by differential interference contrast (DIC) imaging. Only 4 of 408 cells showed partial recovery after bleaching. This represents a ∼1% error rate in our method of scoring completely septated cells. The four cells that did show weak fluorescence recovery out of 408 photobleached cells might represent cells that were in the process of completion of septum assembly in which the division septum resembled a complete septum when visualized by anilin blue staining. Taken together, our data indicated that septation in *cdc16*-116 cells produced attached, but distinct, nucleate and anucleate cells. Thus, *bona fide* anucleate cells could be reliably generated for subsequent investigations of the role of nuclei and SPBs in various cellular processes.

### Dynamic microtubules in anucleate cells

We asked if fission yeast cells could organize the microtubule cytoskeleton in the absence of nuclei and SPBs. There are three major microtubule-organizing centers (MTOCs) in fission yeast–the SPBs, the interphase MTOCs (iMTOCs), and the mitotic equatorial MTOCs (eMTOCs). The SPBs and iMTOCs are associated with the nuclear envelope [22], [23], and the eMTOCs are associated with the actomyosin ring [24]. It has been reported that *cdc16-*116 cells blocked at S phase under non-permissive temperature conditions failed to make eMTOCs [24].

**Figure 3:**
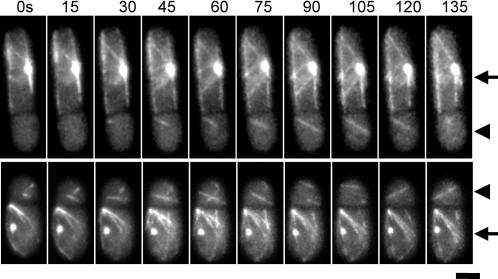
Dynamic cytoplasmic microtubules are present in anucleate compartments. Shown are two time-lapse montages of *cdc16*-116 Pcp1p-GFP cells expressing GFP-Atb2p (α-tubulin). The larger nucleate cells, indicated by arrows, have multiple robust and dynamic microtubule bundles. In contrast, the smaller anucleate cells, indicated by arrowheads, have fewer, yet dynamic microtubules. Scale, 5 µm.

We imaged anucleate cells expressing GFP-α-tubulin to visualize microtubules. To identify the nucleate compartments, the SPB marker Pcp1p-GFP, was also expressed in these cells. In nucleate cells, we observed multiple robust and dynamic microtubule bundles (Figure 3, arrows indicates the compartments with nuclei). These microtubule bundles exhibited growth and shrinkage phases reminiscent of wild-type behavior. Interestingly, dynamic cytoplasmic microtubules were also present in anucleate compartments (Figure 3, arrowheads indicate the anucleate compartments). The behavior of anucleate microtubules resembled that of wild-type microtubules. This result is consistent with the recently published data [8], [9]. Taken together, these experiments indicate that microtubules can self-assemble in the absence of nuclei and SPBs.

### Actomyosin ring assembly in assemble anucleate cells

**Figure 4:**
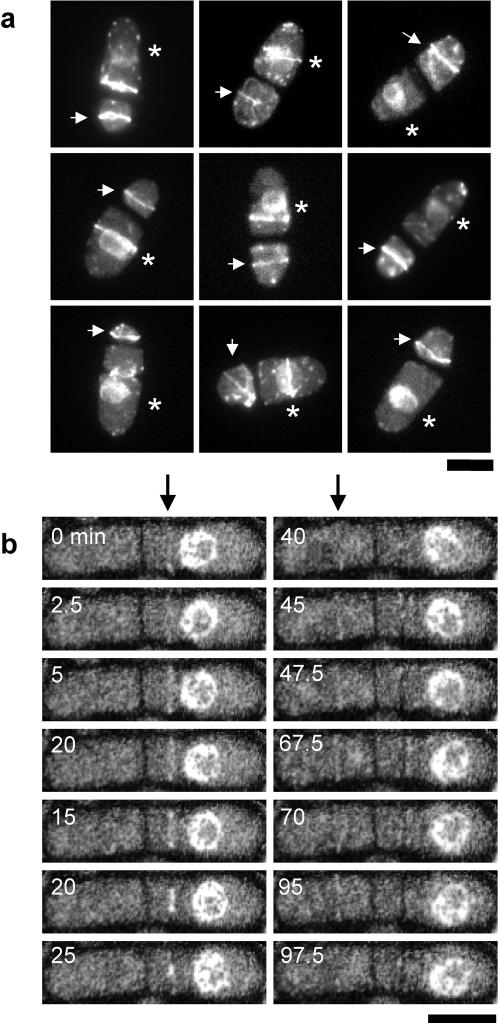
Assembly of cell division structures in anucleate cells. (a) F-actin ring assembly. Shown is a collage of fixed *cdc16*-116 cells expressing the nuclear membrane marker Uch2p-GFP stained with the F-actin specific dye Alexa 488-phalloidin. F-actin rings/cables present in the anucleate compartments are indicated with arrows, while asterisks identify the nucleate compartments. Scale, 10 µm. (b) Actomyosin rings in anucleate cells are unstable and do not constrict. Shown is a time-lapse montage of a live *cdc16*-116 cell expressing the nuclear membrane marker Uch2p-GFP and the actomyosin ring marker Rlc1p-GFP. The connected but distinct cells have a nucleate half and an anucleate half. During time point 0 min to 25 min, a newly formed actomyosin ring was organized in the nucleate cell (arrow, 0 min). This ring underwent complete constriction within ∼30 min. In contrast, although the anucleate cell organized an actomyosin ring (arrow, 40 min), this ring failed to undergo constriction during ∼60 min of observation. Scale, 5 µm.

We then examined if the actomyosin ring can assemble in anucleate cells. *cdc16*-116 cells expressing the nuclear envelope marker Uch2p-GFP [25] were treated as above to generate anucleate cells, and were stained with phalloidin to visualize F-actin (Figure 4a). Strikingly, nucleate and anucleate compartments were found to contain F-actin rings and/or cables. We observed F-actin ring structures in 4.5% (9 out of 200) of anucleate cells. This percentage is statistically higher than the 1% error rate (p<0.05), and we therefore conclude that the F-actin ring structures are indeed organizing in the anucleate cells. Figure 4a shows examples of HU-arrested *cdc16*-116 cells, of which 6 contain rings of F-actin in both the nucleate and anucleate compartments, and 3 contain rings only in the anucleate compartment. F-actin rings in anucleate cells appeared normally organized, although occasionally we also observed cables of F-actin that were not integrated into the F-actin ring (Figure 4a; F-actin rings/cables in the anucleate compartments are indicated with arrows, while asterisks identify the nucleate compartments).

To analyze if the rings organized in anucleate cells also contained type II myosin, *cdc16*-116 cells expressing Uch2p-GFP and Rlc1p-GFP [26], an actomyosin ring protein related to type II myosin regulatory light chains, were treated as above and subjected to live cell imaging. In nucleate cells, actomyosin rings were organized and subsequently underwent constriction (Supplemental movies S1 and S2). These rings in the nucleate compartments did not assemble at the geometric center of the cell as shown previously for the division septa in interphase *cdc16*-116 cells [14] (Supplemental Movie S3, shows several cells assembling acentric rings upon S phase arrest). Consistent with the presence of F-actin rings in anucleate compartments, ∼10% (8 out of 77 cells) of anucleate cells were observed to form rings containing Rlc1p-GFP (Supplemental movies S1 and S2). This percentage is statistically higher than the 1% error rate (p<0.05), and we therefore conclude that the Rlc1p-GFP ring structures are indeed assembled in the anucleate cells. Some of these rings were assembled in the anucleate compartment following assembly of an additional septum in the adjacent nucleate compartment as shown in time lapse images (Supplemental Movies S1 and S2). The rings in anucleate cells also assembled at random locations (Figure 4a). These anucleate-cell rings were qualitatively less intense compared to those in the nucleate cells and failed to constrict. Instead, they disassembled approximately 20–50 minutes after assembly (Figure 4b and supplementary movies S1 and S2). These rings also appeared less robust and might be unstable, given that upon fixation only 9 out of 200 cells were found to contain F-actin rings, whereas in live cell time lapse imaging experiments 8 out of 77 cells were found to be capable of assembling Rlc1p-GFP rings. Thus, although the continued presence of nuclear-associated structures such as SPBs, chromosomes, and/or additional unidentified structures might be important for actomyosin ring constriction and division septum assembly, these structures are strictly not required for ring assembly.

In summary, in this report we have described an efficient method for generation of anucleate fission yeast cells and used this approach to show that the assembly of actomyosin rings and microtubules can occur in the absence of nuclei and SPBs. Thus self-organization mechanisms might play a key role in the organization of cytoskeletal structures such as microtubules and actomyosin rings. We believe this approach should also be useful to the study the role of nuclei and SPBs in the inheritance of mitochondria, endoplasmic reticulum, and Golgi apparatus.

## Supporting Information

Movie S1 Cells undergoing the first division to produce separate nucleate and anucleate halves. Subsequently, the nucleate cells divide again, organizing an actomyosin ring, which constricts and forms a new division septum. In contrast, the anucleate cell organizes an actomyosin ring, which does not constrict.
(2.78 MB MOV)

Movie S2 Cells undergoing the first division to produce separate nucleate and anucleate halves. Subsequently, the nucleate cells divide again, organizing an actomyosin ring, which constricts and forms a new division septum. In contrast, the anucleate cell organizes an actomyosin ring, which does not constrict.
(6.26 MB MOV)

Movie S3 The assembly of rings in interphase arrested cdc16-116 cells. These rings are organized off-center and undergo constriction to divide the cell into two unequal compartments, one of which lacks a nucleus.
(3.03 MB MOV)
